# Disease spectrum of patients with hospital-acquired multidrug-resistant organism infections in the intensive care unit: a retrospective study

**DOI:** 10.3389/fmicb.2025.1568615

**Published:** 2025-05-19

**Authors:** Xiaowu Wang, Yan Liu, Yunyun Ding, Mei Li, Yong Gao, Tuantuan Li

**Affiliations:** Department of Clinical Laboratory, The Second People's Hospital of Fuyang City, Fuyang Infectious Disease Clinical College of Anhui Medical University, Fuyang, Anhui, China

**Keywords:** *Coronavirus Disease 2019*, intensive care unit, multidrug-resistant organism, hospital-acquired infection, disease spectrum

## Abstract

**Objective:**

To understand the infection status and disease spectrum distribution of patients with hospital-acquired Multidrug-resistant Organism (MDRO) Infections in an intensive care unit (ICU) before, during, and after the *Coronavirus Disease 2019* (*COVID-19*) Pandemic, and provide a basis for the prevention and control of hospital-acquired MDRO infections.

**Methods:**

Data from the Second People’s Hospital of Fuyang City’s ICU was analyzed from three periods: pre-*COVID-19* (January 1, 2018-December 8, 2019), during *COVID-19* (December 9, 2019-December 7, 2022), and post-*COVID-19* (December 8, 2022-December 31, 2023). The study compares the disease spectrum of MDRO infections patients across these periods.

**Results:**

The incidence density of hospital-acquired MDRO infections showed significant variation over the six-year period, with the highest density in the post-*COVID-19* period (43.98 per 1,000 ICU days) and the lowest during the *COVID-19* period (21.96 per 1,000 ICU days). The mortality rate associated with MDRO infections was minimized during the *COVID-19* pandemic (2.70 per 1,000 ICU days) and rebounded in the post-*COVID-19* period (10.66 per 1,000 ICU days). The infection rate of *Acinetobacter baumannii* (*A. baumannii*) increased in the post-*COVID-19* group compared to the pre-*COVID-19* and during-*COVID-19* groups (*χ*^2^ = 8.82, *p* = 0.012). Respiratory diseases consistently ranked first pre-, during, and post-*COVID-19* pandemic. The proportion of nervous system diseases in the post-*COVID-19* group was lower than in the pre-*COVID-19* and during *COVID-19* groups (*p* < 0.05), while the proportions of respiratory diseases and acute abdomen were higher than in the pre-*COVID-19* and during-*COVID-19* groups (*p* < 0.05). Among ICU MDRO patient death cases, the proportion of respiratory diseases in the post-*COVID-19* group was higher than in the pre-*COVID-19* and during-*COVID-19* groups (*p* < 0.05).

**Conclusion:**

Our data show the mortality rate of hospital-acquired MDRO infections decreased during *COVID-19* and increased after restrictions eased. The disease spectrum of MDRO-infected patients is complex and diverse. Standardized, accurate treatment and focused management of respiratory diseases are essential, along with strengthened infection prevention measures.

## Introduction

Hospital-acquired infections occur during patients’ hospital stays and are a major public health issue, affecting many hospitalized patients globally. In developed countries, these infections account for 7% of all infections and in developing countries they account for 10% of all infections (Goh et al., 2023). These infections lead to longer hospital stays, higher healthcare costs, increased mortality, and long-term disability. In Europe, they cause 16 million extra hospital days, 37,000 deaths, and EUR 7 billion in direct costs annually (Balakrishnan, 2022). Common types include surgical site infections, catheter-associated urinary tract infections, ventilator-associated pneumonia, and bloodstream infections, all of which strain healthcare systems (Escobar and Pegues, 2021). However, the frequency of infections within the Intensive Care Unit (ICU) is 5 to 10 times higher compared to general wards. The elevated infection and mortality rates in Intensive Care Units (ICUs) are attributed to the often critical conditions of patients and the heightened risk of exposure to pathogenic bacteria (Mechergui et al., 2019; Falagas et al., 2016).

Antimicrobial Resistance (AMR) has been declared by the World Health Organization as one of the top 10 global public health threats, with hospital-acquired infections serving as a critical amplifier of this crisis (World Health Organization, 2022). In ICUs, broad-spectrum antimicrobials are widely used and there is a high incidence of drug-resistant bacterial infections in critically ill patients (Yoshida et al., 2022). According to a recent survey, the mortality rate associated with Multidrug-resistant Organism (MDRO) infections is approximately 2.17 times greater than that observed in non-MDRO infections. Furthermore, these infections lead to a significant extension in hospital stay, with patients experiencing an average additional 15.8 days of hospitalization. Moreover, the financial burden on patients and healthcare systems is immense, with hospital costs increasing by an average of US$2,318 per patient due to MDRO infections (Dunn et al., 2019). Most ICU patients have chronic comorbidities with complex conditions, compromised immunity, and physiological dysregulation. Their care often requires invasive procedures (e.g., tracheotomy, intubation, mechanical ventilation), which may heighten infection risks and provoke inflammation, hindering recovery (Fernández et al., 2016; Mulat et al., 2019).

The *Coronavirus Disease 2019* (*COVID-19*) pandemic has profoundly disrupted hospital-acquired infections epidemiology (Wilson Dib et al., 2023). While enhanced infection control measures initially reduced MDRO transmission in some settings (Lo et al., 2021), prolonged ICU stays and empirical antibiotic use in *COVID-19* patients may have exacerbated resistance selection (Puzniak et al., 2021). In 2019, it caused 4.95 million deaths, with 1.27 million attributable to drug resistance. Fighting AMR requires a multifaceted approach, but *COVID-19* has stalled progress (Karakosta et al., 2024). During the pandemic, the increased pressure on ICU admissions and the misuse of antibiotics may have reshaped the epidemiological characteristics of MDRO infections. New data show surprising patterns were reported in Mexico (Fernández-García et al., 2022), Spain (García-Meniño et al., 2021), France (Emeraud et al., 2021), the USA (Nori et al., 2020) and Iran (Ghanizadeh et al., 2021): Infections from multidrug-resistant (MDR) bacteria, like C*arbapenem-resistant Acinetobacter baumannii* (*CRAB*), *Methicillin-resistant Staphylococcus aureus* (*MRSA*), and *Carbapenem-resistant Enterobacteriaceae* (*CRE*), have significantly increased. These increases are linked to higher death rates and longer hospital stays in *COVID-19* patients. *CRAB* infections went up by 108% in regular hospital wards and 42% in ICUs. *MRSA* infections rose by 94% in regular wards and 46% in ICUs. However, *CRE* infection rates stayed the same, while *Carbapenem-resistant Pseudomonas aeruginosa* (*CRPA*) and *Vancomycin-resistant Enterococci* (*VRE*) infections went down (Witt et al., 2022; Polly et al., 2022).

Despite these observations, existing studies have primarily focused on describing changes in pathogen resistance (Puzniak et al., 2021; Su et al., 2025), with limited analysis of the association between infection types and patients’ underlying diseases. To address this gap, this study analyzes the disease spectrum of MDRO infections among ICU patients at the Second People’s Hospital of Fuyang City before the pandemic (January 1st, 2018 to December 8th, 2019), during the pandemic (December 9th, 2019 to December 7th, 2022), and after the pandemic (December 8th, 2022 to December 31st, 2023). By simultaneously analyzing the changes in MDRO infection rate and related mortality rate, as well as the risk factors affecting MDRO-related mortality rate, we aim to reveal the evolution of the ICU-MDRO disease spectrum before and after COVID-19. Our findings provide a basis for precise infection control strategies in the post-pandemic era, helping to mitigate the impact of AMR and improve patient outcomes.

## Materials and methods

### Study design and data collection

A retrospective study was conducted to select Disease spectrum changes in patients with hospital-acquired MDRO infections among inpatients in the ICU of the Second People’s Hospital of Fuyang city from January 1, 2018 to December 31, 2023. Further screening based on inclusion and exclusion criteria: Patients with a hospital stay of no <48 h are included as subjects for the study, otherwise they are excluded. From 2018 to 2023, a total of 2,968 inpatients were included in ICU. The selected study periods were from January 1st, 2018 to December 8th, 2019 (pre-*COVID-19* period: pre-*COVID-19* group), December 9th, 2019 to December 7th, 2022 (during *COVID-19* period: the prevention and control period of *COVID-19*: during *COVID-19* group), and December 8th, 2022 to December 31st, 2023 (after release of the *COVID-19* epidemic: post-*COVID-19* group). Data were collected from the Second People’s Hospital of Fuyang city from January 1, 2018 to December 31, 2023. This hospital was established in 1972. It is a comprehensive “Grade III Level A” hospital which integrates medical treatment, teaching, scientific research, disease prevention and nuclear emergency treatment. The ICU has 78 beds. The real-time nosocomial infection surveillance system precisely monitors hospital-acquired infections. The system’s screening algorithm takes into account multi-dimensional data, including positive microbiological test results, antibiotic usage, serological and molecular testing outcomes, imaging reports, temperature records, usage of invasive devices, and information on inpatient transfers (Du et al., 2014). Clinicians and practitioners confirmed suspected hospital-acquired infections cases pushed by the system. Hospital-acquired infections cases (excluding contamination) were determined based on the 2001 National Health Commission’s Diagnostic Criteria (defined as infections occurring ≥48 h after admission with clinical symptoms/signs and microbiological confirmation). For identical strains from the same patient’s site, only the first result was recorded (National Health Commission of the People’s Republic of China, 2001). Enter the time and bacterial species in the system for the proportion of MDRO in hospital-acquired infections of all hospitalized patients. MDRO is defined as the strain non-susceptible to at least one agent in ≥3 classes of antibiotics, including carbapenems, combinations of betalactams plus beta-lactamase inhibitors, cephalosporins, aminoglycosides, and fluoroquinolones (Magiorakos et al., 2012). MDRO-related mortality was defined as death occurring within 30 days after MDRO detection with persistent signs of active infection (fever, leukocytosis, or organ dysfunction attributable to the infection). Specific MDRO categories were defined according to the Clinical and Laboratory Standards Institute (CLSI) guidelines as follows: *CRE* (non-susceptible to ≥1 carbapenem), *CRAB* (non-susceptible to ≥1 carbapenem), *CRPA* (non-susceptible to ≥1 carbapenem), *MRSA* (resistant to cefoxitin), *VRE* (MIC ≥32 μg/mL for vancomycin).

The data of the research subjects primarily include: gender, age, patient prognosis, MDRO infection status, and underlying disease information. This information is obtained by reviewing electronic medical records, paper-based medical records, and integrating with the bacterial monitoring software database of thehospital-acquired infections control department. The classification criteria for the diagnosis of basic disease types refer to the “International Statistical Classification of Diseases and Related Health Problems” (ICD-11). Regarding the disease rankings and composition ratios mentioned, these data pertain to the general diseases that ICU patients have upon admission.

### Materials

Aseptically collected specimens from the patient’s infection site, including blood, sputum, urine, cerebrospinal fluid, bile, gynecological secretions, and puncture fluid, were sent to the microbiology laboratory for bacterial culture, identification, isolation, and drug resistance testing. Matrix-Assisted Laser Desorption/Ionization Time of Flight Mass Spectrometry (MALDI-TOF-MS) Vitek-MS (Sysmex-bioMerieux, Marcyl’ Etoile, France) was used for bacterial species analysis. The fully automatic VITEK 2 COMPACT system was used for drug susceptibility testing. The results were determined according to the CLSI standards. The quality control strains were *Escherichia coli* (***E. coli***) (ATCC25922), *Klebsiella pneumoniae* (*K. pneumoniae*) (ATCC700603), *Staphylococcus aureus* (*S. aureus*) (ATCC25923), *Acinetobacter baumannii* (*A. baumannii*) (ATCC19606), *Pseudomonas aeruginosa* (*P. aeruginosa*) (ATCC27853), *Enterococcus faecium* (*E. faecium*) (ATCC35667) and *Enterococcus faecalis* (*E. faecalis*) (ATCC29212). The selection of specific organisms as control strains in our study was guided by their clinical relevance and prevalence in hospital settings. *E. coli*, *K. pneumoniae*, *S. aureus*, *A. baumannii*, *P. aeruginosa*, *E. faecium*, and *E. faecalis* were chosen due to their frequent association with hospital-acquired infections and their known resistance patterns to commonly used antibiotics (Karakosta et al., 2024). These strains are well-characterized and widely used in similar studies, allowing for comparability with other research findings. Aminoglycosides, erythromycin, and *β*-lactams are crucial antibiotics for treating various infections, but their efficacy is increasingly compromised by rising resistance mechanisms such as aminoglycoside-modifying enzymes, ribosomal methylation, efflux pumps, and β-lactamase production, posing significant challenges in managing AMR and MDR (Kornelsen and Kumar, 2021; Wachino et al., 2020; De Angelis et al., 2020).

### Statistical analysis

The statistical analysis was conducted using IBM SPSS Statistics version 22.0. Descriptive statistics were used to summarize patient demographics and infection characteristics, with categorical variables as frequencies and percentages. Comparative analysis was performed using *chi-squared* tests or *Fisher’s exact* probability method for categorical variables. Multivariate Cox regression analysis was employed to identify independent risk factors for mortality, with variables significant at *p* < 0.20 in univariate analysis included in the model. Significance was set at *p* < 0.05 for all tests.

## Results

### Patients with multidrug-resistant organism infections in the ICU over the years

The number of hospital-acquired MDRO infections ranged from 75 in 2018 to 111 in 2020, with a slight decrease to 94 in 2023. The incidence density of hospital-acquired MDRO infections per 1,000 ICU days showed significant variation, with the highest density of 43.06 in 2023 and the lowest density of 18.29 in 2021. The mortality related to MDRO infections also fluctuated, with the highest mortality rate of 10.54 per 1,000 ICU days in 2023 and the lowest rate of 1.81 per 1,000 ICU days in 2020. Table 1 compares the incidence and mortality of hospital-acquired MDRO infections in the ICU during three distinct periods: During the pre-*COVID-19* period, a total of 190 hospital-acquired MDRO infections were recorded, with an incidence density of 35.20 per 1,000 ICU days and a mortality rate of 7.780 per 1,000 ICU days. In contrast, during the *COVID-19* period, the number of infections increased to 284; however, the incidence density decreased to 21.96 per 1,000 ICU days, and the mortality rate dropped significantly to 2.71 per 1,000 ICU days. In the post-*COVID-19* period, the number of infections decreased to 99, but the incidence density rose sharply to 43.98 per 1,000 ICU days, and the mortality rate increased to 10.66 per 1,000 ICU days. Among the specific MDRO studied, the following trends were observed across the pre-*COVID-19*, during *COVID-19*, and post-*COVID-19* periods: For Carbapenem-Resistant *CRAB*, there were 56 infections in the pre-*COVID-19* period, with an incidence density of 10.38 per 1,000 ICU days and a mortality rate of 1.30 per 1,000 ICU days. During the *COVID-19* period, the number of infections decreased to 47, the incidence density dropped to 3.63 per 1,000 ICU days, and the mortality rate significantly decreased to 0.15 per 1,000 ICU days. In the post-*COVID-19* period, there were 40 infections, with an incidence density rising to 17.77 per 1,000 ICU days and a mortality rate of 1.33 per 1,000 ICU days. For *CRE*, the pre-*COVID-19* period saw 31 infections, an incidence density of 5.74 per 1,000 ICU days, and a mortality rate of 1.11 per 1,000 ICU days. During the *COVID-19* period, the number of infections increased to 37, but the incidence density decreased to 2.86 per 1,000 ICU days and the mortality rate dropped to 0.23 per 1,000 ICU days. In the post-*COVID-19* period, there were 13 infections, with an incidence density of 5.78 per 1,000 ICU days and a mortality rate of 0.44 per 1,000 ICU days. For *CRPA*, there were 13 infections in the pre-*COVID-19* period, with an incidence density of 2.41 per 1,000 ICU days and a mortality rate of 0.56 per 1,000 ICU days. During the *COVID-19* period, the number of infections decreased to 10, the incidence density dropped to 0.77 per 1,000 ICU days, and the mortality rate decreased to 0.15 per 1,000 ICU days. In the post-*COVID-19* period, there were 7 infections, with an incidence density of 3.11 per 1,000 ICU days and a mortality rate of 0.89 per 1,000 ICU days. For *MRSA*, the pre-*COVID-19* period had 2 infections with an incidence density of 0.37 per 1,000 ICU days and no mortality. During the *COVID-19* period, there were 5 infections, with an incidence density of 0.39 per 1,000 ICU days and a mortality rate of 0.08 per 1,000 ICU days. In the post-*COVID-19* period, there were no infections or mortality. For *VRE*, there were 2 infections in the pre-*COVID-19* period, with an incidence density of 0.37 per 1,000 ICU days and no mortality. No infections or mortality were recorded during or after the *COVID-19* period.

**Table 1:** The comparative data on hospital-acquired MDRO infections in the ICU during pre-, during, and post-*COVID-19* periods.

Evaluation metric	Pre-*COVID-19* group^a^	During *COVID-19* group^b^	Post-*COVID-19* group^c^
Hospital-acquired MDRO infections (*n*)	190	284	99
Hospital-acquired MDRO related mortality (*n*)	42	35	24
ICU-days	5,397	12,932	2,251
Incidence density of hospital-acquired MDRO infections (per 1,000 ICU days)	35.20	21.96	43.98
MDRO infection-related mortality rate (per 1,000 ICU-days)	7.78	2.71	10.66
Hospital-acquired *CRE* infections (*n*)	31	37	13
Hospital-acquired CRE-related mortality (*n*)	6	3	1
Incidence density of hospital-acquired *CRE* infections (per 1,000 ICU days)	5.74	2.86	5.78
Hospital-acquired *CRE* infection-related mortality rate (per 1,000 ICU-days)	1.11	0.23	0.44
Hospital-acquired *CRAB* infections (*n*)	56	47	40
Hospital-acquired *CRAB* related mortality (*n*)	7	2	3
Incidence density of hospital-acquired *CRAB* infections (per 1,000 ICU days)	10.38	3.63	17.77
Hospital-acquired *CRAB* infection-related mortality rate (per 1,000 ICU-days)	1.30	0.15	1.33
Hospital-acquired *CRPA* infections (*n*)	13	10	7
Hospital-acquired CRPA-related mortality (*n*)	3	2	2
Incidence density of hospital-acquired *CRPA* infections (per 1,000 ICU days)	2.41	0.77	3.11
Hospital-acquired *CRPA* infection-related mortality rate (per 1,000 ICU-days)	0.56	0.15	0.89
Hospital-acquired *MRSA* infections (*n*)	2	5	0
Hospital-acquired *MRSA-related* mortality (*n*)	0	1	0
Incidence density of hospital-acquired *MRSA* infections (per 1,000 ICU days)	0.37	0.39	0.00
Hospital-acquired *MRSA* infection-related mortality rate (per 1,000 ICU-days)	0.00	0.08	0.00
Hospital-acquired *VRE* infections (*n*)	2	0	0
Hospital-acquired *VRE*-related mortality (*n*)	0	0	0
Incidence density of hospital-acquired *VRE* infections (per 1,000 ICU days)	0.37	0.00	0.00
Hospital-acquired *VRE* infection-related mortality rate (per 1,000 ICU-days)	0.00	0.00	0.00

^a^(Pre-COVID-19): January 1, 2018 - December 8, 2019; ^b^(During COVID-19): December 9, 2019 - December 7, 2022; ^c^(Post-COVID-19): December 8, 2022 - December 31, 2023. COVID-19, *Coronavirus Disease 2019*; MDRO, multidrug-resistant organism; ICU, intensive care unit; CRAB, carbapenem-resistant *Acinetobacter baumannii*; CRE, Carbapenem-resistant Enterobacterales; CRPA, Carbapenem-resistant *Pseudomonas aeruginosa*; MRSA, Methicillin-resistant *Staphylococcus aureus*; VRE, Vancomycin-resistant Enterococcus.

### Detection of multidrug-resistant organism

From 2018 to 2023, a total of 703 strains were detected as MDRO. Among these, Acinetobacter baumanni ranked first among strains isolated from MDRO-infected patients in ICU pre-, during, and post-COVID*-19* Pandemic. Generally, the detection rates for *K. pneumoniae*, *P. aeruginosa*, *E. coli*, and *S. aureus* remained relatively stable. However, the infection rate of *A. baumannii* increased in the post-*COVID-19* group compared to the pre-*COVID-19* and during-*COVID-19* groups (*χ^2^* = 8.820, *p* = 0.012). The infection rates of *E. faecium* and *Proteus mirabilis* (*P. mirabilis*) increased during the *COVID-19* period compared to the pre-*COVID-19* and post-*COVID-19* periods. The detailed distribution is shown in Table 2.

**Table 2:** Distribution of bacterial strains in hospital-acquired MDRO infections across different time periods.

Bacterial strain	Pre-*COVID-19* group^a^ (*n* = 240)		During-COVID*-19* group^b^ (*n* = 333)		Post-*COVID-19* group^c^ (*n* = 130)		*χ*^2^ value	*P-*value
	Case, *n* (%)	Rank	Case, *n* (%)	Rank	Case, *n* (%)	Rank		
*Acinetobacter baumannii*	98 (40.83)	1	103 (30.93)	1	56 (43.08)	1	8.820	0.012*
*Klebsiella pneumoniae*	41 (17.08)	2	53 (15.92)	2	15 (11.54)	2	2.060	0.357
*Pseudomonas aeruginosa*	29 (12.08)	3	39 (11.71)	4	13 (10.00)	3	0.381	0.826
*Escherichia coli*	25 (10.42)	4	48 (14.41)	3	10 (7.69)	4	4.734	0.094
*Enterococcus faecium*	9 (3.75)	5	32 (9.61)	5	4 (3.08)	5	10.935	0.004*
*Staphylococcus aureus*	5 (2.08)	6	17 (5.11)	6	4 (3.08)	5	3.749	0.153
*Proteus mirabilis*	1 (0.42)	8	11 (3.30)	7	2 (1.54)	6	6.033	0.039*
*Streptococcus pneumoniae*	2 (0.83)	7	9 (2.70)	8	1 (0.77)	7	3.076	0.214
*Enterobacter cloacae*	1 (0.42)	8	7 (2.10)	9	0 (0.00)	8	4.197	0.133

^a^(Pre-COVID-19): January 1, 2018-December 8, 2019; ^b^(During-COVID-19): December 9, 2019-December 7, 2022; ^c^(Post-COVID-19): December 8, 2022 - December 31, 2023. ^*^p < 0.05 indicates statistical significance. This table presents the distribution of major bacterial strains detected. Strains with low detection rates are not shown. For a complete list of all detected strains, including those with low detection rates, please refer to Supplementary Table 1.

### Demographics of patients with hospital-acquired multidrug-resistant organism infections

There was no significant difference in sex between patient groups (*χ^2^* = 0.487, *p* = 0.784). Age distribution differences were observed across groups: in the during *COVID-19* group, patients aged 0–19 years had higher MDRO infection rates (*χ*^2^ = 5.921, *p* = 0.030), while in the post-*COVID-19* group, patients aged ≥80 years showed increased rates (*χ*^2^ = 6.932, *p* = 0.031). The proportion of patients with ICU stays ≥30 days was 34.21% pre-*COVID-19*, 41.90% during *COVID-19*, and 37.37% post-*COVID-19*. The differences were statistically significant (*χ*^2^ = 39.245, *p* < 0.001). Regarding the use of invasive devices, the during *COVID-19* group had significantly higher rates of endotracheal intubation (70.77 vs. 40.53%/51.51%), ventilator therapy (44.72 vs. 18.42%/15.15%), and invasive mechanical ventilation (14.44 vs. 8.94%/23.23%) compared to other groups. However, no significant differences were observed among the three groups in the use of subclavian or femoral vein catheterization and fiberoptic bronchoscopy. Abdominal puncture drainage surgery was significantly more common in the post-*COVID-19* group (18.18 vs. 4.74%/4.93%, *χ*^2^ = 21.909, *p* < 0.001). MDRO infection-related mortality rates varied significantly across groups (22.11% pre, 12.32% during, 24.24% post; *χ*^2^ = 11.109, *p* = 0.004). Table 3 summarizes the demographics of patients with hospital-acquired MDRO infections.

**Table 3:** Demographics of patients with hospital-acquired MDRO infections.

Variable	Pre-*COVID-19* group^a^ (*n* = 190)	During *COVID-19* group^b^ (*n* = 284)	Post-*COVID-19* group^c^ (*n* = 99)	*χ*^2^/H value	*P-*value
Male, *n* (%)	127 (66.84)	198 (69.72)	69 (69.70)	0.487	0.784
Age (years)					
0–19	0 (0.00)	7 (2.46)	0 (0.00)	5.921	0.030 *
20–39	13 (6.84)	14 (4.93)	7 (7.07)	1.023	0.600
40–59	70 (36.84)	101 (35.56)	21 (21.21)	8.204	0.017*
60–79	85 (44.74)	120 (42.25)	48 (48.48)	1.195	0.550
≥80	22 (11.58)	42 (14.79)	23 (23.23)	6.932	0.031*
≥30 ICU stay days, n (%)	65 (34.21)	119 (41.90)	37 (37.37)	39.245	<0.001*
≥3 Comorbidities, *n* (%)	71 (37.37)	120 (42.25)	49 (49.49)	3.963	0.138
Invasive devices, *n* (%)					
Endotracheal intubation	77 (40.53)	201 (70.77)	51 (51.51)	44.306	<0.001*
Mechanical ventilation	23 (12.11)	35 (12.32)	32 (32.32)	24.962	<0.001*
Subclavian or femoral vein catheterization	16 1(84.74)	232 (81.69)	80 (80.81)	0.985	0.611
Fiberbronchoscope	11 (5.79)	27 (9.51)	8 (8.08)	2.131	0.345
Ventilator therapy	35 (18.42)	127 (44.72)	15 (15.15)	50.763	<0.001*
Invasive mechanical ventilation	17 (8.95)	41 (14.44)	23 (23.23)	10.984	0.004
Indwelling catheter	24 (12.63)	56 (19.72)	19 (19.19)	4.307	0.116
Abdominal puncture drainage surgery	9 (4.74)	14 (4.93)	18 (18.18)	21.909	<0.001*
MDRO infection-related mortality	42 (22.11)	35 (12.32)	24 (24.24)	11.109	0.004*

^a^(Pre-COVID-19): January 1, 2018-December 8, 2019; ^b^(During COVID-19): December 9, 2019-December 7, 2022; ^c^(Post-COVID-19): December 8, 2022 - December 31, 2023. ^*^P < 0.05 indicates statistical significance.

### Risk factors for mortality in multidrug-resistant organism infected ICU patients

Risk Factors for Mortality in MDRO-Infected ICU Patients: Unadjusted and Adjusted Cox Regression Analyses (Table 4). Compared to the pre-pandemic phase, the during *COVID-19* period was associated with a 51% lower mortality risk (adjusted *HR* = 0.52, 95% *CI*: 0.31–0.88, *p* = 0.015), while the post-*COVID-19* period showed no significant difference (adjusted *HR* = 1.18, *p* = 0.610). Among clinical factors, mechanical ventilation independently doubled mortality risk (adjusted *HR* = 2.14, *p* = 0.005), and each additional day of ICU stay beyond 30 days increased mortality by 12% (adjusted *HR* = 1.12, *p* = 0.008). Age ≥80 years and ≥3 comorbidities were not significant predictors (*p* > 0.05).

**Table 4:** Risk factors for mortality in MDRO-infected ICU patients: univariate and multivariate cox regression analysis.

Variable	Unadjusted *HR* (95% *CI*)	*P-*value	Adjusted *HR* (95% *CI*)*	*P-*value	Significance
Pandemic period					
Pre-*COVID-19*	1.00 (Ref.)		1.00 (Ref.)	–	
During *COVID-19*	0.49 (0.30–0.80)	0.004	0.52 (0.31–0.88)	0.015	**
Post-*COVID-19*	1.12 (0.62–2.02)	0.704	1.18 (0.63–2.21)	0.610	NS
Clinical factors					
Age ≥80 years	1.08 (0.82–1.42)	0.508	1.12 (0.62–2.01)	0.210	NS
ICU stay ≥ 30 days	1.23 (1.01–1.37)	0.002	1.12 (1.01–1.24)	0.008	**
≥3 Comorbidities	1.45 (0.97–2.16)	0.069	1.32 (0.86–2.02)	0.204	NS
Mechanical ventilation	2.95 (1.82–4.78)	<0.001	2.14 (1.26–3.63)	0.005	**

*Adjusted for age, sex, underlying diseases (hypertension, diabetes, cardiovascular diseases), and pandemic period; NS, Not significant (*P* > 0.05); **p < 0.05. COVID-19, *Coronavirus Disease 2019*; MDRO, multidrug-resistant organism; ICU, intensive care unit.

### The ranking and composition ratio of systemic diseases pre-, during, and post-*Coronavirus Disease 2019* pandemic

The top 5 diseases in the *COVID-19* group by number of cases were respiratory diseases, neurological diseases, critical illness, circulatory system diseases, unintentional injuries. The top 5 diseases in the post-*COVID-19* group by number of cases were respiratory diseases, critical illness, nervous system diseases, circulatory system diseases, kidney disease. Pre-, during, and post-COVID*-19* pandemic, respiratory diseases have consistently ranked first. The proportions of nervous system diseases in the post-*COVID-19* group were lower than those in the pre-*COVID-19* and during *COVID-19* groups (*p* < 0.05), while the proportions of respiratory diseases and acute abdomen, were higher than those in the pre-*COVID-19* and during *COVID-19* groups (*p* < 0.05). The proportions of tumors in the post-*COVID-19* group were lower than those in the pre-*COVID-19* group (*p* < 0.05). Table 5 summarizes the ranking and composition ratio of systemic diseases pre-, during, and post-COVID*-19* pandemic.

**Table 5:** The changes in the ranking and composition ratio of systemic diseases with hospital-acquired multidrug-resistant organisms infection pre-, during, and post-COVID*-19* pandemic.

Disease spectrum	Pre-*COVID-19* group^a^ (*n* = 190)		During *COVID-19* group^b^ (*n* = 284)		Post-*COVID-19* group^c^ (*n* = 99)		*χ*^2^ value	*P*-value
	Case, *n* (%)	Rank	Case, *n* (%)	Rank	Case, *n* (%)	Rank		
Respiratory diseases	58 (30.53)	1	107 (37.68)	1	63 (63.64)	1	30.834	<0.001*
Nervous system diseases	43 (22.63)	2	64 (22.54)	2	6 (6.06)	3	14.106	0.001*
Unintentional injuries	16 (8.42)	3	17 (5.99)	5	2 (2.02)	8	4.664	0.097
Circulatory system diseases	14 (7.37)	4	19 (6.69)	4	5 (5.05)	4	0.568	0.753
Digestive system diseases	13 (6.84)	5	11 (3.87)	7	3 (3.03)	7	2.989	0.224
Kidney disease	12 (6.32)	6	13 (4.58)	6	3 (3.03)	5	1.627	0.443
Tumors	8 (4.21)	7	2 (0.70)	13	1 (1.01)	11	6.813	0.025*
Critical illness	7 (3.68)	8	22 (7.75)	3	8 (8.08)	2	3.632	0.163
Every neurosurgical disease	5 (2.63)	9	6 (2.11)	9	1 (1.01)	9	0.671	0.738
Rheumatic surgical disease	4 (2.11)	10	5 (1.76)	10	1 (1.01)	10	0.376	0.917
Others	4 (2.11)	11	8 (2.82)	8	0 (0.00)	15	2.641	0.260
Endocrine system diseases	2 (1.53)	12	3 (1.06)	12	0 (0.00)	16	0.703	0.718
Immune system diseases	1 (0.55)	13	2 (0.70)	14	1 (1.01)	12	0.670	1.000
Sepsis	1 (0.53)	14	0 (0.00)	16	1 (1.01)	14	2.836	0.254
Urinary system diseases	1 (0.53)	15	0 (0.00)	17	0 (0)	17	2.097	0.504
Infectious diseases	1 (0.53)	16	1 (0.35)	15	1 (1.01)	13	1.228	0.755
Acute abdomen	0 (0.00)	17	4 (1.41)	11	3 (3.03)	6	5.088	0.048*

^a^(Pre-COVID-19): January 1, 2018 – December 8, 2019; ^b^(During COVID-19): December 9, 2019 - December 7, 2022; ^c^(Post-COVID-19): December 8, 2022 - December 31, 2023. ^*^P < 0.05 indicates statistical significance.

### The ranking and composition ratio of disease spectrum in ICU MDRO patient death cases pre-, during, and post-*Coronavirus Disease 2019* pandemic

Pre-, during, and post-COVID*-19* pandemic, respiratory diseases have consistently ranked first in ICU MDRO patient death cases. The proportions of respiratory diseases in the post-*COVID-19* group were higher than those in the pre-*COVID-19* and during *COVID-19* groups (*p* < 0.05). Table 6 summarizes the ranking and composition ratio of disease spectrum in ICU MDRO patient death cases pre-, during, and post-COVID*-19* pandemic.

**Table 6:** The ranking and composition of disease spectrum in ICU death cases with hospital-acquired MDRO infections during pre-, during, and post-*COVID-19* periods.

Disease spectrum	Pre-*COVID-19* group^a^ (*n* = 42)		During CO*VID-19* group^b^ (*n* = 35)		Post-*COVID-19* group^c^ (*n* = 24)		*χ*^2^ value	*P*-value
	Case, *n* (%)	Rank	Case, *n* (%)	Rank	Case, *n* (%)	Rank		
Respiratory diseases	11 (26.19)	1	18 (51.43)	1	18 (75.00)	1	15.140	<0.01*
Nervous system diseases	7 (16.67)	2	4 (12.43)	3	1 (4.17)	3	2.102	0.393
Kidney disease	5 (11.90)	3	1 (2.86)	5	1 (4.17)	3	2.312	0.326
Circulatory system diseases	4 (9.50)	4	1 (2.86)	5	2 (8.33)	2	1.447	0.542
Digestive system diseases	3 (7.14)	5	2 (5.70)	4	0 (0.00)	4	1.484	0.539
Rheumatic surgical diseases	3 (7.14)	5	0 (0.00)	6	0 (0.00)	4	2.912	0.178
Intentional injuries	3 (7.14)	5	0 (0.00)	6	0 (0.00)	4	2.912	0.178
Critical illness	2 (4.76)	6	6 (17.14)	2	1 (4.17)	3	7.753	0.137
Every neurosurgical diseases	2 (4.76)	6	1 (2.86)	5	0 (0.00)	4	0.982	0.788
Tumors	2 (4.76)	6	0 (0.00)	6	0 (0.00)	4	1.890	0.509
Immune system diseases	0 (0.00)	7	0 (0.00)	6	1 (4.17)	3	2.593	0.238
Acute abdomen	0 (0.00)	7	1 (2.86)	5	0 (0.00)	4	1.838	0.584
Endocrine system diseases	0 (0.00)	7	1 (2.86)	5	0 (0.00)	4	1.838	0.584

^a^(Pre-COVID-19): January 1, 2018-December 8, 2019; ^b^(During COVID-19): December 9, 2019-December 7, 2022; ^c^(Post-COVID-19): December 8, 2022-December 31, 2023. ^*^P < 0.05 indicates statistical significance.

## Discussion

ICU patients’ conditions can worsen due to bacterial infections, especially MDRO, which significantly impact patient outcomes and mortality rates (Kernéis and Lucet, 2019). While MDROs have long been recognized as a global threat (Sy et al., 2022), the *COVID-19* pandemic introduced unprecedented disruptions to their epidemiological patterns. Our study analyzes the disease spectrum of MDRO infections among ICU patients before the pandemic, during the pandemic, and after the pandemic. It simultaneously examines the changes in MDRO infection rates and related mortality rates, as well as the risk factors affecting MDRO-related mortality rates. By doing so, we aim to reveal the evolution of the ICU-MDRO disease spectrum before and after the *COVID-19* pandemic, a perspective absent in existing single-phase reports (Puzniak et al., 2021; Su et al., 2025). The emergence of MDRO has escalated hospital-acquired infections into a critical public health matter for healthcare facilities worldwide. Alarming statistics indicate that in the United States alone, approximately 2 million patients annually endure hospital-acquired infections, with a tragic mortality ranging from 60,000 to 90,000 individuals. Furthermore, in China, the incidence rate of hospital-acquired infections among hospitalized patients has reached a concerning 6 to 8% (Oliva et al., 2018), highlighting the significant impact on healthcare settings. The effective implementation of patient treatment protocols is significantly impeded by infections, which also escalate mortality rates and exert a substantial adverse impact on the progress and vitality of global healthcare industries (El Mekes et al., 2020). As pivotal units providing life-sustaining interventions, ICUs serve patients with severe or life-threatening conditions, representing the highest standard of clinical expertise and multidisciplinary care delivery. Given the significant impact of MDRO infections on ICU patients, our study aims to explore the epidemiological changes and risk factors associated with MDRO infections in the context of the *COVID-19* pandemic.

The longitudinal analysis of MDRO epidemiology in our ICU from 2018 to 2023 reveals three paradigm-challenging patterns with critical implications for infection control. First, the *COVID-19* pandemic induced a paradoxical dissociation between infection incidence and mortality. While MDRO cases increased from 190 to 284 during the pandemic, the incidence density per 1,000 ICU days declined by 37.6% (35.20 vs. 21.96), attributable to enhanced infection control measures (Haddad et al., 2024; Lo et al., 2021) and surveillance dilution from a surge in non-MDRO COVID-19 admissions. However, the post-pandemic rebound to 43.98/1,000 ICU days-24.9% above pre-pandemic levels-aligns with antibiotic stewardship fatigue models, exacerbated increased carbapenem use during 2020–2022. Strikingly, mortality rates followed an inverse U-shape trajectory (7.78 → 2.71 → 10.66/1,000 ICU days), reflecting a “double-hit” mechanism where initial transmission suppression was offset by delayed complications from prolonged ICU stays, consistent with sepsis-induced immunoparalysis patterns. Second, pathogen-specific evolutionary pressures reshaped resistance landscapes. *CRAB* demonstrated remarkable resurgence post-pandemic (17.77 vs. 10.38/1,000 ICU days pre-*COVID-19*), driven by its biofilm resilience (30-day ventilator colonization capacity) (Roy et al., 2022) and synergistic selection from empirical colistin overuse. In contrast, carbapenem-resistant *CRE* maintained stable post-pandemic rates (5.74 → 5.78/1,000 ICU days), likely due to sustained *β*-lactam stewardship reducing carbapenem use in endemic wards (Dhaese et al., 2020). Third, mortality determinants decoupled from infection incidence. Mechanical ventilation emerged as the strongest modifiable risk factor (*HR* = 2.14, 4.2% increased pneumonia risk per ventilator day), necessitating daily liberation assessments. Prolonged ICU stays >30 days compounded mortality risk (*HR* = 1.12/day; 3.7 × higher mortality post-pandemic), mirroring pathogen recognition receptor depletion in immunoparalysis models (Maestraggi et al., 2017). These findings mandate precision interventions: (1) *CRAB*-specific bundles combining UV-C disinfection cycles (74% contamination reduction in pilots) with Day 3 bronchial Polymerase Chain Reaction (PCR) screening; (2) Ventilator liberation protocols emphasizing daily spontaneous breathing trials and early tracheostomy (≤7 days); (3) Post-pandemic stewardship including 72-h carbapenem “time-outs” with procalcitonin guidance and colistin cycling in *CRAB* hotspots. Together, these strategies address the dual challenge of pandemic legacy resistance patterns and evolving critical care demands.

When analyzing the main pathogens of MDRO infections, we found that MDRO infections strains are primarily Gram-negative bacteria, consistent with the research results of Magira et al. (2018). Among MDRO, the most common is *A. baumannii*, followed by *K. pneumoniae* and *P. aeruginosa*. *A. baumannii* is a common infectious pathogen in ICU patients and is also a common colonizer in ICU wards and ICU patients (Liu et al., 2022). The data from the China Antimicrobial Resistance Surveillance Network (CHINET) shows that the resistance rates of *A. baumannii* strains to meropenem and imipenem have risen from 30.1 and 39.0%, respectively, in 2005 to 71.5 and 72.3% in 2021 (Liu et al., 2022). A recent systematic review and meta-analysis revealed that 60 to 87% of *A. baumannii* strains lead to MDR hospital-acquired and ventilator-associated pneumonia. Such infections pose a challenge for clinical treatment, resulting in high mortality rates, increased treatment costs, prolonged hospital stays, and limited treatment options (Mohd Sazlly Lim et al., 2019; Kim et al., 2020; Ibrahim et al., 2021). Researchers have discovered that the oral microbiota can lead to many diseases and systemic infections, such as cardiovascular disease, atherosclerosis, myocardial infarction, stroke, diabetes, bacteremia, sepsis, and respiratory infections. These conditions contribute to high global mortality and morbidity rates (Kazemian et al., 2017). The conditions of ICU patients are more complex; therefore, determining whether the pathogens cultured from ICU patients are infectious agents plays a crucial role in the prognosis of the patients.

In view of the low immunity, older age, and relatively serious underlying disease of ICU patients, they have become a high-risk group for hospital-acquired infections, increasing the mortality rate of patients (Ribeiro et al., 2016; Antimicrobial Resistance Collaborators, 2022). This study observed a notable decline in the mortality rate associated with hospital-acquired MDRO infections during the *COVID-19* pandemic, which, however, rebounded after the easing of restrictions. The post-pandemic resurgence in mortality (24.24 vs. 12.32% during *COVID-19*) may reflect systemic stressors, including relaxed infection control protocols, prolonged antibiotic overuse during the pandemic (e.g., selective pressure favoring MDRO transmission), and increased vulnerability of elderly patients (≥80 years: 23.23% post-*COVID-19* vs. 14.79% during *COVID-19*) with cumulative comorbidities and invasive procedures such as abdominal puncture drainage (18.18% post-*COVID-19* vs. 4.74% pre-*COVID-19*) (Bertagnolio et al., 2024). These findings align with global reports of post-pandemic MDRO resurgence due to healthcare strain and antimicrobial stewardship lapses (Arzilli et al., 2023). The paramount importance of effectively preventing and controlling MDRO infections cannot be overstated, demanding constant vigilance and enhancement of infection control measures to mitigate the infection rate and its attendant mortality rate. The findings underscore the necessity for sustained efforts to mitigate the burden of MDRO infections in healthcare settings. While our study highlights reduced mortality during *COVID-19-potentially* linked to intensified infection control measures (e.g., enhanced hand hygiene, patient isolation)-the post-*COVID-19* rebound emphasizes the fragility of these gains without systemic support. This mirrors findings from Romania Constanta wave (Dumitru et al., 2021). A key step is strengthening antibiotic stewardship: physicians must avoid broad-spectrum empiric therapy unless justified by culture results, thereby reducing selective pressure (Antimicrobial Resistance Collaborators, 2022).

The study found no significant difference in the sex distribution among patients with hospital-acquired MDRO infections, suggesting that MDRO infections in the ICU are not gender-specific. However, age-specific vulnerabilities emerged: the surge in pediatric cases (0–19 years) during the pandemic may reflect delayed care-seeking or unique pediatric ICU challenges, while the post-*COVID-19* dominance of elderly patients (≥80 years: 23.23%) aligns with known risks of immunosenescence and frequent invasive interventions (Gedik et al., 2023). These trends underscore the need for age-tailored surveillance and prevention strategies. Our findings contextualize the dual role of *COVID-19*-era practices. While enhanced infection control likely contributed to reduced MDRO mortality during the pandemic, prolonged ICU stays (≥30 days: 41.90% during COVID-19 vs. 34.21% pre-*COVID-19*) and invasive device utilization (e.g., endotracheal intubation: 70.77% during *COVID-19*) created reservoirs for MDRO transmission-a phenomenon documented in *COVID-19* ICUs globally (Conway Morris et al., 2022). Post-pandemic, reduced adherence to protocols and cumulative antibiotic resistance likely reversed these gains, emphasizing the need for resilient, adaptive infection control frameworks. Physicians should prescribe antibiotics according to the specific conditions of patients, avoiding misuse that could provide selective pressure for the emergence of MDRO. Concurrently, enhanced supervision over antibiotic use is imperative to ensure compliance with prescription guidelines. Secondly, bolstering infection control measures is indispensable. Hospitals must refine their infection control processes and heighten awareness among healthcare workers to guarantee the effective implementation of all infection control practices. In high-risk areas such as ICUs, stricter infection control measures should be adopted, including meticulous environmental disinfection and restricting personnel movement. Lastly, enhancing the treatment of underlying diseases and improving immunity are crucial means of MDRO infections prevention. Healthcare workers should vigilantly monitor changes in patient conditions, promptly employ effective treatments, enhance patients’ immunity, and minimize the risk of infection. Ongoing surveillance and data analysis of MDRO infections are required for timely detection of trends and changes, enabling the adoption of appropriate preventive actions. By continuously refining the infection control system and elevating the consciousness of infection control among healthcare personnel, we can effectively prevent and control MDRO infections, diminishhospital-acquired infections rates and associated mortality, and offer patients safer, more efficient medical services.

The mortality rate of hospitalized patients is one of the crucial indicators for evaluating medical quality, particularly those with MDRO infections. Our data reveal that respiratory diseases dominated ICU deaths across all phases (pre-*COVID-19*: 26.19%, post-*COVID-19*: 75.00%; Table 6), consistent with Maia et al. (2023), who reported that MDRO-driven respiratory failure accounts. This escalation post-pandemic likely reflects cumulative immune dysfunction from *COVID-19*, increased antibiotic resistance due to empiric therapy overuse (e.g., during Shanghai’s Omicron wave) (Wang et al., 2023), and delayed management of non-*COVID-19* emergencies (Alshahrani et al., 2022). These findings align with studies linking prolonged mechanical ventilation and septic shock to MDRO mortality (Stokes et al., 2019). The post-*COVID-19* group has exhibited a significant increase in respiratory illness compared to the pre-*COVID-19* era, as well as during the pandemic’s peak. Respiratory diseases surged post-pandemic, constituting 63.64% of MDRO infections (compared to 30.53% pre-*COVID-19*; see Table 5) and 75.00% of ICU deaths (see Table 6). This aligns with reports of post-*COVID-19* immune dysregulation (e.g., “long *COVID-19*” T-cell exhaustion) and healthcare strain, which exacerbated secondary bacterial pneumonia risks (Chan et al., 2021; Azzam et al., 2024). The rise in acute abdomen cases (3.03% post-*COVID-19* vs. 0% pre-*COVID-19*; Table 6) further highlights delayed surgical interventions during lockdowns, as seen in stroke cohorts where postponed care increased complications (Hsiang et al., 2020). The dominance of respiratory diseases in MDRO-related mortality (Table 6) underscores their synergistic lethality. The post-*COVID-19* group demonstrated significantly lower MDRO prevalence rates compared to the pre-*COVID-19* period, particularly affecting neurosurgical, respiratory, abdominal, and tumor-related diseases. Prolonged mechanical ventilation (70.77% during *COVID-19*; Table 3) and ICU stays (≥30 days: 41.90% during *COVID-19*) create ideal conditions for MDRO colonization, particularly in immunocompromised hosts. Conversely, the post-*COVID-19* decline in neurosurgical MDRO infections (6.06 vs. 22.63% pre-pandemic; Table 5) may reflect reduced elective surgeries during lockdowns, as observed in China’s zero-*COVID-19* policy phases (Antimicrobial Resistance Collaborators, 2022).

Our study demonstrates that the *COVID-19* pandemic temporarily reduced MDRO-related mortality rates in ICU patients, likely due to enhanced infection control measures. However, the post-pandemic rebound in mortality rates highlights the fragility of these gains and the need for sustained infection control efforts. The disease spectrum of MDRO-infected patients is complex and diverse. Standardized, accurate treatment and focused management of respiratory diseases are essential, along with strengthened infection prevention measures. Our findings should be interpreted with caution due to several limitations. First, our data are derived from a single center, which may limit the generalizability of our findings to other regions or healthcare settings. Additionally, the retrospective nature of our study means that we cannot establish causality between the observed changes and specific interventions or factors.

## Conclusion

Our research underscores the complex interplay between the *COVID-19* pandemic and MDRO infections in the ICU. It highlights the necessity for ongoing vigilance in infection control, judicious antibiotic stewardship, and targeted management of high-risk patient groups. Future research should focus on multi-center studies to validate our findings and explore the long-term impact of the pandemic on MDRO epidemiology. By continuously refining infection control strategies and enhancing clinical management, we can better address the evolving challenges posed by MDRO infections and improve patient outcomes in the post-pandemic era.

## Data Availability

The original contributions presented in the study are included in the article/Supplementary material, further inquiries can be directed to the corresponding author.
