# Supplementary material for: Disease spectrum of patients with hospital-acquired multidrug-resistant organism infections in the intensive care unit: a retrospective study
Source: Front Microbiol. 2025 May 19;16:1568615. doi: 10.3389/fmicb.2025.1568615 (PMC12127312; doi:10.3389/fmicb.2025.1568615)
Supplement: Supplementary file 1 [file Data_Sheet_1.doc]

Additional Table 1: Distribution of Low-Detection-Rate Bacterial Strains in Hospital-Acquired MDRO Infections Across Different Time Periods

| bacterial strain | pre-*COVID-19* group^a^ | During- *COVID-19* group^b^ | Post-*COVID-19* group^c^ |
| --- | --- | --- | --- |
| Serratia marcescens | 1 | 0 | 1 |
| Ornithinibacillus californiensis | 1 | 0 | 1 |
| Sphingomonas paucimobilis | 0 | 0 | 1 |
| Aeromonas hydrophila | 0 | 0 | 0 |
| Acinetobacter pittii | 1 | 0 | 1 |
| Acinetobacter lwoffii | 1 | 0 | 1 |
| Acinetobacter calcoaceticus | 1 | 1 | 1 |
| Achromobacter xylosoxidans | 2 | 1 | 0 |
| Ralstonia mannitolilytica | 1 | 0 | 1 |
| Haemophilus influenzae | 1 | 0 | 3 |
| Haemophilus influenzae type b | 1 | 2 | 1 |
| Providencia rettgeri | 2 | 1 | 1 |
| Acinetobacter junii | 1 | 0 | 2 |
| Citrobacter fruendtii | 1 | 2 | 1 |
| Citrobacter amalonaticus | 1 | 1 | 2 |
| Aeromonas caviae | 1 | 1 | 2 |
| Proteus vulgaris | 1 | 0 | 1 |
| Citrobacter koseri | 2 | 0 | 0 |
| Citrobacter braakii | 0 | 0 | 0 |
| Serratia rubidaea | 1 | 0 | 0 |
| Aeromonas sobria | 1 | 0 | 0 |
| Streptococcus dysgalactiae | 1 | 0 | 0 |
| Streptococcus gordonii | 1 | 0 | 0 |
| Proteus penneri | 2 | 1 | 1 |
| Staphylococcus simulans | 1 | 1 | 0 |
| Staphylococcus schleiferi | 0 | 1 | 1 |
| Streptococcus parahaemolyticus | 0 | 0 | 1 |
| Elizabethkingia meningoseptica | 2 | 0 | 0 |
| burkholderia cepacia | 1 | 0 | 1 |
| Stenotrophomonas maltophilia | 0 | 2 | 1 |

Footnotes: a (Pre-*COVID-19*): January 1, 2018–December 8, 2019; b (During *COVID-19*): December 9, 2019-December 7, 2022; c (Post-*COVID-19*): December 8, 2022-December 31, 2023.
